# D-dimer levels during and after anticoagulation withdrawal in patients with venous thromboembolism treated with non-vitamin K anticoagulants

**DOI:** 10.1371/journal.pone.0219751

**Published:** 2019-07-16

**Authors:** Cristina Legnani, Ida Martinelli, Gualtiero Palareti, Alessandro Ciavarella, Daniela Poli, Walter Ageno, Sophie Testa, Daniela Mastroiacovo, Maurizio Ciammaichella, Eugenio Bucherini, Nicola Mumoli, Benilde Cosmi

**Affiliations:** 1 Fondazione Arianna Anticoagulazione, Bologna, Italy; 2 Fondazione Ca’ Granda (Istituto di Ricovero e Cura a Carattere Scientifico) - Ospedale Maggiore Policlinico - A. Bianchi Bonomi Hemophilia and Thrombosis Center, Milano, Italy; 3 Malattie Aterotrombotiche - Azienda Ospedaliero Universitaria Careggi, Firenze, Italy; 4 Degenza Breve Internistica e Centro Trombosi ed Emostasi - Azienda Socio Sanitaria Territoriale dei Sette Laghi, Varese, Italy; 5 Centro Emostasi e Trombosi - Laboratorio Analisi chimico-cliniche e microbiologiche - Azienda Socio Sanitaria Territoriale di Cremona, Cremona, Italy; 6 Unità Operativa Semplice Dipartimentale di Angiologia e Diagnostica Vascolare - Ospedale Civile S.S. Filippo e Nicola, Avezzano, L’Aquila, Italy; 7 Unità Operativa Complessa di Medicina d’Urgenza - Azienda Ospedaliera S. Giovanni Addolorata, Roma, Italy; 8 Struttura Semplice Dipartimentale - Medicina Vascolare – Angiologia – Ospedale Civile Faenza, Faenza, Ravenna, Italy; 9 Unità Operativa Complessa di Medicina Interna - Ospedale Fornaroli, Magenta, Milan, Italy; 10 Unità Operativa Angiologia e Malattie della Coagulazione - Azienda Ospedaliero Universitaria di Bologna - Policlinico S. Orsola - Malpighi, Bologna, Italy; Institut d’Investigacions Biomediques de Barcelona, SPAIN

## Abstract

**Background:**

D-dimer levels measured during and after vitamin K antagonist withdrawal may be used in clinical practice to assess the individual risk of recurrent venous thromboembolism. Currently, direct oral anticoagulants (DOACs) are frequently used in venous thromboembolism treatment; however, their pharmacokinetics and pharmacodynamics characteristics are completely different than vitamin K antagonists. The present study aimed at comparing the results of D-dimer levels during and after anticoagulation withdrawal in patients with venous thromboembolism treated with DOACs or warfarin.

**Material and methods:**

D-dimer levels were measured in 527 patients (“cases”) during DOACs treatment (T0) and after 15 (T15), 30 (T30), 60 (T60) and 90 (T90) days after their discontinuation and in 527 patients (“controls”) enrolled in the DULCIS study (all treated with warfarin), matched for sex, age (+/-3 y), type of D-dimer assay and site of venous thromboembolism. Both cases and controls received anticoagulant treatment after a first venous thromboembolism event that was unprovoked or associated with weak risk factors.

**Results:**

The rate of positive D-dimer results was significantly higher in cases than in controls at T0 (10.8% vs 5.1%, p = 0.002) and at T30 (18.8% vs 11.8%, p = 0.019), as well as at the other time-points, though not statistically significant.

**Conclusion:**

D-dimer levels during and after stopping an anticoagulant treatment for a venous thromboembolism episode differ between patients treated with a DOAC than in those treated with warfarin. Specifically designed prospective studies are warranted to reassess the use of D-dimer as predictor of the risk of recurrent venous thromboembolism in patients treated with DOACs.

## Introduction

Venous thromboembolism (VTE), that includes deep vein thrombosis (DVT) and/or pulmonary embolism (PE), is a frequent, severe, acute disease. After the first episode, VTE has a risk to recur that increases sharply after anticoagulation is stopped, especially in patients with unprovoked events. Anticoagulant therapy is absolutely necessary to treat the acute phase of the disease, to avoid early complications, and it is also indicated in the long term to avoid recurrences. Clinical studies have shown that three months of initial treatment reduce the risk of recurrence over a shorter period of anticoagulation [1]. However, whatever the duration of the anticoagulant treatment thereafter, the protective benefit fades after anticoagulation is stopped and the risk of recurrence increases again in higher risk patients [2–5]. Clinical practice guidelines therefore recommend at least 3 months of anticoagulation after a first unprovoked VTE; and in patients at high risk of recurrent VTE and non-high risk of bleeding, indefinite (potentially lifelong) treatment is recommended [6]. The same guidelines suggest stratifying patients according to risk of recurrent VTE, using D-dimer testing, a fibrin degradation product and a marker of coagulation activation. Positive D-dimer results identify patients with a high risk for recurrence (because of a persistent pro-thrombotic tendency) in whom indefinite anticoagulation is justified [7]. Conversely, negative D-dimer may indicate patients at low risk of recurrence, in whom extended anticoagulation may be unjustified [8]. D-dimer use for this proposal has been investigated and confirmed by many clinical studies. In most of the studies, D-dimer level was measured from 3 to 4 weeks after anticoagulation was stopped; in some studies D-dimer were measured serially during and at different times after anticoagulation was stopped [9–11]; finally, in one study measurement was made only during anticoagulant therapy [12]. It is noteworthy to stress that all the currently available studies on D-dimer measurement to assess the risk of recurrent VTE events included only patients receiving vitamin K antagonists (VKAs, in most cases warfarin) as oral anticoagulant drug. In recent years, different oral anticoagulant drugs (direct oral anticoagulants, DOACs), have increasingly been used for VTE treatment instead of VKAs. Currently, it is not known whether D-dimer levels may have the same pattern during and after anticoagulation is stopped in patients treated with DOACs, drugs that have pharmacokinetics and pharmacodynamics characteristics completely different from VKAs.

The present study aimed at assessing the results of D-dimer levels during and after anticoagulation is stopped in VTE patients treated with DOACs, in comparison with the D-dimer changes recorded in patients treated with warfarin who were included in the DULCIS study, in which serial D-dimer measurements were used to decide on management of anticoagulant therapy duration in patients after unprovoked VTE events [11].

## Materials and methods

### Patient populations

The present is a retrospective, case-control study, focusing on D-dimer level changes during and after anticoagulant treatment was stopped in patients who received either DOACs or warfarin for VTE therapy. The present study does not address the predictive value for VTE recurrence of D-dimer testing. The “cases” were VTE patients treated with DOACs who were evaluated to manage the duration of anticoagulant treatment (see below). The “controls” were matched patients selected among the patients included in the DULCIS study who were treated with warfarin [11]. Both cases and controls received anticoagulant treatment after a first VTE episode, that could be unprovoked or associated with weak risk factors that included: minor general, laparoscopic, or arthroscopic surgery; pregnancy or puerperium; hormonal contraceptive or replacement therapy; long travel (> 6 hours); minor trauma, leg injury, reduced mobility or hospitalization in a medical ward. The DULCIS patients followed a protocol based on serial D-dimer measurements at the following time intervals: during anticoagulation (T0), and 15 (T15), 30 (T30), 60 (T60), and 90 (T90) days after its suspension. As soon as the first positive D-dimer was recorded, patients were recommended to continue or resume anticoagulant therapy (at the time of the study only VKAs were available for chronic therapy). Those with persistently negative D-dimer permanently interrupted anticoagulation. D-dimer measurements were performed in the participant centers by using the quantitative assay customarily adopted in the centers and results are available in the central database.

To realize this study, we asked some Italian centers, which routinely adopted a serial D-dimer measurement procedure to decide the duration of anticoagulant therapy in VTE patients treated with DOACs, if they were willing to centralize to the coordinating center the characteristics of the patients (in anonymous way), and the quantitative results of D-dimer measurements. A positive answer was obtained from 9 centers, which contributed to the study by sending data from examined patients (“cases”).

Patients treated with warfarin were selected among patients enrolled in the DULCIS study (registered at clinicaltrials.gov as #NCT00954395) and those treated with DOACs were included in the START-Register (#NCT02219984). The DULCIS study and the START-Register have been approved by the Ethical Committee of the coordinating center (Azienda Ospedaliero-Universitaria, Policlinico S. Orsola-Malpighi, Bologna, Italy). The local Ethics Committees approval was then obtained by all participating centers. All patients provided informed written consent to have data from their medical records used in research. Data were fully anonymized before transferring to the central analysis office.

### Matching procedure

The cases (subjects treated with DOACs), gathered from the 9 participating centers, were listed onto an electronic page and printed to show only the fields to be used to select the appropriate matching controls (treated with warfarin as VKA), following this order: the commercial assay used to perform D-dimer measurements (see Table 1), sex, age (+- 3 years), and type of index event (DVT, DVT + PE, isolated PE). The control subjects were selected in the coordinating center from the general DULCIS database. The fields relevant for the selection, were copied onto an electronic page and printed following the order detailed above. An author (C.L.) of the study, unaware of all the remaining information contained in the database, was responsible for selecting a matched control for each case. We decided to do not match cases and controls for the duration of previous anticoagulant treatment since in the DULCIS study it was demonstrated that this characteristic did not influence the prevalence of positive or negative D-dimer results [11].

**Table 1:** Baseline characteristics of patients treated with DOACs (cases) or warfarin (controls).

Characteristic	Cases n = 527	Controls n = 527	P
**Male sex**, n. (%)	323 (61.3)	323 (61.3)	1.000
**Age** (y), median (IQ)	58 (46–71)	60 (46–71)	0.482
**Age > 70 y**, n. (%)	141 (26.8)	141 (26.8)	1.000
**Type of VTE**, n. (%)			
*Proximal DVT (no PE)*	291 (55.2)	289 (54.8)	0.896
*Proximal DVT with symptomatic PE*	128 (24.3)	125 (23.7)	0.820
*Isolated PE*	108 (20.5)	113 (21.5)	0.690
**Presence and type of risk factor**, n. (%)			
*No risk factors (unprovoked)*	365 (69.3)	385 (73.1)	0.173
*Weak risk factors*	162 (30.7)	142 (26.9)	
**Commercial D-dimer assays** (results in FEU), n.			
*Innovance D-dimer (Siemens)*	28	28	
*Sta Liatest D-Di (Diagnostica Stago)*	241	241	
*VIDAS D-Dimer (bioMerieux)*	2	2	
**Commercial D-dimer assays** (results in D-dimer units), n.			
*HemosIL D-Dimer HS (Werfen)*	256	256	
**Type of DOAC**, n.			
*Apixaban 2*.*5 mg/2 die*	11		
*Apixaban 5*.*0 mg/2 die*	92		
*Dabigatran 110 mg/2 die*	1		
*Dabigatran 150 mg/2 die*	41		
*Edoxaban 30 mg/die*	3		
*Edoxaban 60 mg/die*	27		
*Rivaroxaban 15 mg/die*	6		
*Rivaroxaban 20 mg/die*	346		

DVT = deep vein thrombosis; DOACs = direct oral anticoagulants; FEU = fibrinogen equivalent units; PE = pulmonary embolism; VTE = venous thromboembolism

### The definition of positive/negative D-dimer results

Differently than in the DULCIS study, in the present report, positive or negative D-dimer results were established using the cut-off values indicated for VTE exclusion by the commercial D-dimer assays. Results were therefore considered positive when > 230 ng/ml for the assays expressing results as D-dimer units and > 500 ng/ml for those using fibrinogen equivalent units (FEU) as expression of results. D-dimer results were considered positive when above the mentioned cut-off values.

### Statistical analysis

Continuous variables are presented as median and interquartile range. The Mann-Whitney U-test and the Chi-square test (Yates corrected) were used for group comparison, as appropriate; all p values less than 0.05 were considered to indicate statistical significance. For statistical analysis the SPSS software package (SPSS, Chicago, III, USA) and the GraphPad Software (San Diego, CA, USA) were used.

## Results

We collected the results of 527 VTE patients (“cases”), who were receiving a treatment with a DOAC. These patients had started a serial D-dimer measurement procedure to decide whether to continue or resume anticoagulation if and when D-dimer assay resulted positive. Among them, 66.8% were receiving Rivaroxaban (the first DOAC approved for this indication in our country), 19.5% Apixaban, 8.0% Dabigatran and 5.7% Edoxaban; 21/527 were treated with the corresponding low dose of DOACs. An equal number of VTE patients, receiving warfarin (“controls”), were selected by means of the matching procedure detailed in Methods section. The characteristics of the two cohorts are shown in Table 1.

**Fig 1:**
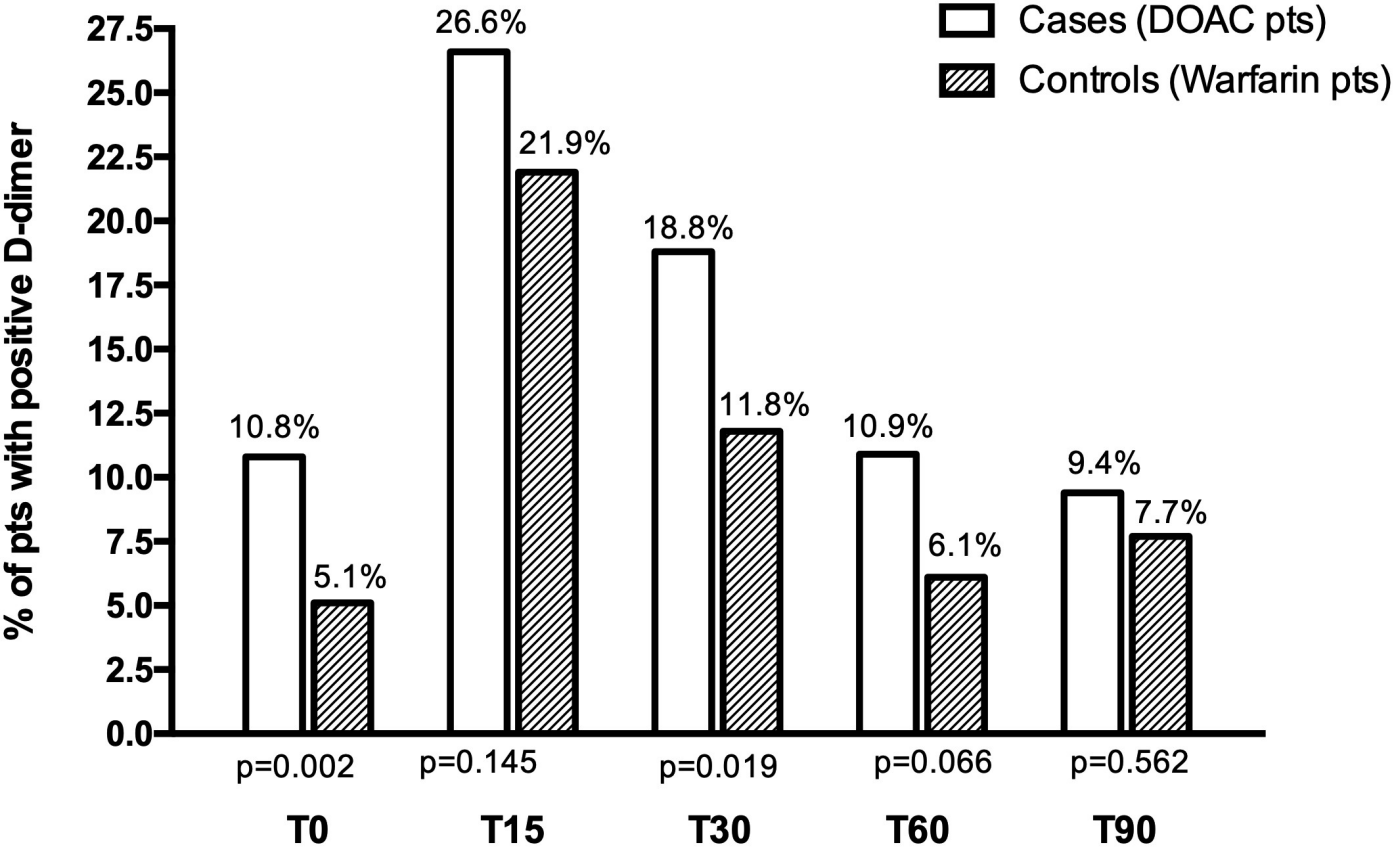
Prevalence of positive D-dimer at different time-points in patients treated with DOACs (cases) or warfarin (controls). T0 = during anticoagulation; T15, T30, T60 and T90 = 15, 30, 60 and 90 days after anticoagulation withdrawal, respectively.

As shown in Fig 1, the rate of positive D-dimer results was higher in cases than in controls at T0: 10.8% vs 5.1%, respectively, p = 0.002, and at T30: 18,8% vs 11.8%, p = 0.019. Though always higher in cases, the rates were not significantly different in the other time-point measurements.

When the results obtained at T0 (during treatment) were analyzed in details, it was found (Table 2) that significantly higher rates of positive di D-dimer in cases were detected in young subjects (aged 70 years or less), when the index event was unprovoked, was represented by DVT with or without PE, and when the assays used for D-dimer measurement were those using FEU to express results.

**Table 2:** Prevalence of patients with positive D-dimer measured during treatment (T0) in patients treated with DOACs (cases) or warfarin (controls).

	Patients with positive D-dimer (%) during treatment (T0)		P
	Cases	Controls	
All patients	10.8	5.1	0.002
Patients after exclusion of those treated with low-dose DOACs (n. 506)	10.2	5.1	0.002
Males	9.4	4.5	0.029
Females	12.9	6.1	0.036
Age <= 70 years	8.3	1.3	<0.0001
Age > 70 years	21.5	15.4	0.334
Unprovoked events	11.1	5.3	0.033
WRF-associated events	10.1	4.4	0.082
DVT+/-PE	11.0	5.5	0.010
Isolated PE	9.7	3.7	0.110
Assay reporting results as FEU	21.8	6.6	0.0001
Assays reporting results as D-dimer units	7.1	3.6	0.084

DVT = deep vein thrombosis; DOACs = direct oral anticoagulants; FEU = fibrinogen equivalent units; PE = pulmonary embolism; VTE = venous thromboembolism; WRF = weak risk factor

The rates of positive D-dimer at different time-points resulted high in the small group of cases (21 subjects) treated with reduced DOAC doses (T0 = 18.2%, T15 = 40.0%, T30 = 60.0%, T90 = 50.0%). As expected, the age of these subjects was significantly higher than that of patients who received standard doses (median age 68 y; IQR 52–79.5 y; vs 58 y; IQR 46–71, respectively; p = 0.039). After exclusion of the cases treated with reduced DOAC doses, the difference at T0 (during anticoagulation) between positive D-dimer rates in standard dose DOAC-treated cases and controls did not change (Table 2).

No differences in the rates of positive D-dimer at the different time-points were found between patients treated with once or twice per day DOAC dosing (Table 3).

**Table 3:** Prevalence of positive D-dimer at different time-points in patients treated with once (Rivaraxaban and Edoxaban) or twice (Dabigatran and Apixaban) per day dosing. T0 = during anticoagulation; T15, T30, T60 and T90 = 15, 30, 60 and 90 days after anticoagulation withdrawal, respectively.

	Patients with positive D-dimer (%)		P
	Once per day DOACs n = 382	Twice per day DOACs n = 145	
T0	9.0%	15.1%	0.453
T15	24.4%	33.1%	0.087
T30	17.7%	20.2%	0.609
T60	10.0%	10.0%	0.511
T90	10.1%	7.1%	0.633

## Discussion

This study investigates, for the first time, the course of D-dimer levels in patients with VTE treated with DOACs, measured during and after stopping anticoagulation. The rate of positive D-dimer assessed during anticoagulation was significantly higher in patients treated with DOACs than warfarin. After anticoagulation was stopped, the rates of positive D-dimer in the subsequent time measurements were still higher in DOACs than in warfarin, though difference reached a statistical significance only at the 30 days measurement control. While the higher prevalence of positive D-dimer during treatment with DOACs than with warfarin can be interpreted as a result of the completely different mechanism of action on coagulation balance of the two types of anticoagulant agents, more difficult is to hypothesize an explanation for the different prevalence of positive results that was detected at a later time after anticoagulation was stopped. It can be surmised that, similarly to what occurs in some warfarin-treated patients whose D-dimer measurements become positive at different time after the treatment is stopped, even in DOAC-treated patients laboratory signs of hypercoagulability may appear at a later time after treatment withdrawal, and more frequently than in warfarin-treated patients due to a putative higher baseline condition of activated coagulation. The high prevalence of positive D-dimer results among the few patients who were treated with reduced DOAC doses, was likely due to the selection of elderly patients (in whom D-dimer levels are in general increased) for the reduced dose-treatment. Focusing on results obtained during anticoagulation (T0, Table 2), positive D-dimer results were significantly more frequent in DOAC-treated patients than in controls when patients were younger, had unprovoked events and when the index event was DVT with/without PE; furthermore, the difference was particularly relevant when D-dimer levels were measured using assays reporting results as FEU. This finding suggests a possible influence of the various D-dimer assays on results obtained in patients who are anticoagulated with warfarin or DOACs and deserves further assessment.

The presence of high D-dimer plasma levels is in general considered a sign of hypercoagulability [13]. D-dimer assay has several indications in clinical laboratory, particularly in the diagnostic process to exclude VTE in symptomatic patients. More recently, D-dimer testing is also being used to assess the individual risk of VTE recurrence, to recommend extended anticoagulation in patients in whom positive test results indicate a high risk of recurrence [7, 8, 11]. Our results suggest that more patients treated with DOACs would receive indication for extended anticoagulation than those treated with warfarin and that a sign of hypercoagulability during treatment is more frequent in the former than in the latter patients.

The most important limitation of this study is that we do not have data about comparison of VTE recurrences according to D-dimer levels during and after stopping anticoagulant treatment, since we did not collect follow-up in patients treated with DOACs. Other important limitations are: the retrospective design, the indirect comparison with a warfarin-treated control group derived from a different study, the potential persisting effects of factors not included in the matching procedure, the imbalance between patients receiving the different DOAC drugs (with a high prevalence of rivaroxaban, the first DOAC admitted in our country for VTE indication), the relatively not high number of examined patients, and the non-centralized D-dimer determination, that was performed in the participating centers using different commercial assays.

The present study does not show any data regarding the risk of recurrent VTE after stopping anticoagulant therapy with either DOACs or warfarin according to the results of D-dimer measurements. Our results should, therefore, only be considered as hypothesis generating for specifically designed, prospective studies to assess the presence and potential clinical effects of signs of hypercoagulability during DOAC treatment in VTE patients; they also indicate that the use of D-dimer to predict the risk of VTE recurrence should be reassessed in patients treated with DOACs.
